# Factors influencing HPV vaccine implementation in South Asia: a scoping review protocol

**DOI:** 10.1186/s13643-023-02415-x

**Published:** 2024-01-05

**Authors:** Priyobrat Rajkhowa, Prachi Pundir, Sherize Merlin Dsouza, Divya Sussana Patil, Prakash Narayanan, Helmut Brand

**Affiliations:** 1 Department of Health Policy, Prasanna School of Public Health (PSPH), Manipal Academy of Higher Education (MAHE), Manipal, Udupi, Karnataka PIN 576104 India; 2 Department of International Health, Care and Public Health Research Institute – CAPHRI, Faculty of Health Medicine and Life Sciences, Maastricht University, Maastricht, The Netherlands; 3 The George Institute for Global Health, 308, Third Floor, Elegance Tower, Plot No. 8, Jasola District Centre New Delhi, 110025 India; 4 Transdisciplinary Centre for Qualitative Methods (TCQM), Department of Health Information, Prasanna School of Public Health (PSPH), Manipal Academy of Higher Education (MAHE), Manipal, India; 5 Public Health Evidence South Asia, Prasanna School of Public Health (PSPH), Manipal Academy of Higher Education (MAHE), Manipal, Karnataka India

**Keywords:** Cervical cancer, HPV vaccine, Human papillomavirus, Implementation

## Abstract

**Introduction:**

The HPV vaccine is characterized by its significant effectiveness in preventing the occurrence of cervical cancer. However, the South Asian countries face multiple challenges in implementing the human papillomavirus vaccine (HPV) at scale. Implementation of human papillomavirus vaccination for eliminating cervical cancer necessitates investigating the factors that impact the health system of these nations. Hence, this review will map the evidence on factors influencing the scaling up of human papillomavirus vaccination in South Asia.

**Methods:**

The proposed scoping review will follow the steps given by Arksey and O’Malley and Levac et al*.* The search approach will follow McGowan et al. (14) evidence-based manual for Peer Analysis of Electronic Search Strategies (PRESS 2015) for systematic searches. Using a comprehensive search, the literature from 2006 onward will be identified from PubMed, CINAHL, EMBASE, Web of Science, and Scopus. The search strategy will include terms relating to the HPV vaccine and implementation. A predefined criterion for the inclusion and exclusion of studies will be adopted by three review authors independently to determine the eligible studies. The results will be narratively synthesized and examined in addition to being quantitatively presented to provide an outline. The review will be presented per the “Preferred Reporting Items for Systematic Reviews and Meta-analyses extension for scoping review (PRISMA-ScR)” guidelines.

**Conclusions/discussion:**

The evaluation is anticipated to map the barriers and enablers influencing the rollout of the human papillomavirus vaccine. Lessons learned from the South Asian countries, where the vaccine has been implemented, may contribute to aiding the implementation of the vaccine in countries with similar health systems in an effective manner.

**Systematic review registration:**

The protocol was prospectively registered on the “open science framework”. The registration DOI is https://doi.org/10.17605/OSF.IO/T5SW9.

## Introduction

Cervical cancer is a major global public health issue, resulting in premature disability and mortality. The GLOBCON Report of 2020 highlights a consistent upward trajectory in the incidence of cervical cancer, reaching a staggering global count of 604,127 cases and contributing to 341,831 fatalities [1]. Remarkably, more than half of these cases emerge within the Asia-Oceania region, with the Southeast and South-central Asia subregions exhibiting the highest rates of both incidence and mortality [2, 3]. This epidemiological distribution underscores the critical nature of the issue that requires prevention and interventions.

Low- and middle-income countries (LMICs) lack organized cervical cancer screening and human papillomavirus (HPV) vaccination programs, which account for approximately 90% of cervical cancer cases [4]. Given the gravity of this challenge, the World Health Organization (WHO) has devised a multifaceted strategy termed the “triple-intervention coverag” approach. This strategy entails implementation of HPV vaccination as one of the key strategies for eliminating cervical cancer particularly within LMICs [5]. Notably, the relevance of this approach extends to South Asian nations, as a significant portion of these countries falls within the LMIC classification [6].

Furthermore, the World Health Organization (WHO) has outlined various guidelines to assist nations in introducing the vaccine into their national immunization programs [7]. Despite these efforts, the extent of vaccine coverage in a majority of South Asian countries remains insufficient [8]. Only a limited subset of nations in South Asia, namely Bhutan, Maldives, and Sri Lanka, have successfully incorporated the vaccine into their routine national immunization schedules, as documented by PATH in 2022 [8]. The suboptimal implementation of the vaccine can be ascribed to an array of factors encompassing socio-cultural, healthcare system, and political [9]. In the South Asian context, India, encountered ethical quandaries during HPV vaccine demonstration initiatives, leading to the suspension of the vaccination program [10]. Similarly, these nations face multifaceted impediments in the expansion of vaccination endeavors. The absence of thorough analysis and comprehensive consideration of potential influential elements during the planning phase might contribute to a range of challenges in attaining desired health outcomes [11]. Therefore, it holds significance to comprehend the influencing elements affecting the execution of HPV vaccination and to derive insights from past occurrences in South Asia pertaining to the introduction of the HPV vaccine. This evidence is crucial for addressing concerns in future efforts to improve implementation and scaling up of HPV vaccination and make informed decisions and develop plans to make the HPV vaccination effective in South Asia. Ultimately, this aids in achieving the World Health Organization’s objective of eliminating cervical cancer in this region by 2030.

## Methodology

The scoping review protocol is developed by following the framework given by Arksey and O'Malley and Levac et al. [12, 13]. This review will be outlined as per the Preferred Reporting Item for Scoping Reviews (PRISMA-ScR) guidelines. We used “PRISMA-P: Preferred Reporting Items for Systematic review and Meta-Analysis Protocols 2015 checklist” to describe minimal recommended objects to address this scoping review protocol and is given in Annexure 1.

The following steps will be used to conduct this review:

### Stage 1: Identifying the review question

To come up with the review question, the team went through a process of brainstorming and refining ideas. A literature search on HPV vaccine implementation in LMICs resulted in a broad research question, followed by refinement of the research question.

1. What are the enablers and barriers to scaling up the HPV vaccination program in South Asia?

The study’s objectives were developed in response to the preceding research question. The objectives of the scoping review are as follows:

1. To identify factors influencing implementation of HPV vaccination in South Asia.
2. Outline the lessons learnt and highlight the empirical evidence from South Asian nations on implementing the HPV vaccination at scale.

PCC (Population, Concept, Context) format, according to the JBI manual for evidence synthesis 2020, has been adopted for developing the research question [14]. (Table 1)

**Table 1:** PCC framework for developing the research question

Population (P)	Adolescent females: These include female age between 9 and 13 years, suggested by the WHO [15]
Concept (C)	Factors associated with the mass implementation/introduction/uptake/scale up of the HPV vaccine. The factors can be both facilitators or barriers to HPV vaccine implementation
Context (C)	South Asia (Afghanistan, Bangladesh, Bhutan, India, Maldives Nepal, Pakistan, Sri Lanka)

### Stage 2: Identifying relevant studies

We will use the “evidence-based manual for Peer Analysis of Electronic Search Strategies (PRESS 2015)” for systematic searches McGowan et al. [16] to inform the search approach [16]. The review question will be divided into concepts and keywords within each concept will be identified. The eligible studies will be searched by using a combination of the following keywords in the databases “HPV vaccine,” “HPV vaccination,” “implementation,” South Asia,” “Gardasil,” “Cervarix,” “Human papillomavirus vaccin*,” “Uptake,” “Scaleup,” and the names of countries within South Asia. Boolean operators AND/OR will be used to combine keywords. Search will be limited to English language articles only. A thorough search of numerous databases, including PubMed, CINAHL, EMBASE, Web of Science, and Scopus, will be used to find relevant literature. An overview of the electronic search for one of the selected databases (PubMed) as a reference have been enclosed in Annexure 2. More potentially qualifying articles will be retrieved by searching the reference lists of included papers. Data will be managed using Rayyan software [17].

### Stage 3: Study selection

All identified citations will be gathered, uploaded into Rayyan, and duplicates will be eliminated after the search. All retrieved titles-abstracts will be screened by three reviewers, who will also assess the papers for eligibility using the pre-defined inclusion criterion. A fourth reader or an independent opinion may be requested if the reviewers are unsure about the study’s eligibility to be included in the analysis. The authors will agree on potentially relevant research through collected screening consensus, and the full text will be retrieved for review through agreement. The full texts of the studies included after the first stage (title and abstract screening) will be screened by three reviewers independently. When sources of evidence in the complete text do not match the inclusion criteria, the reviewer will note and describe the reasons for exclusion. The included studies will be considered for data analysis. The final scoping review will include an extensive report on the search findings and the procedure for included studies, which will be presented as a flowchart using the PRISMA 2020 flow diagram [18]*.* The studies will be selected based on two comprehensive processes given below:

1. For the initial screening, there will be limits based on country. Countries from South Asia only will be included.
2. Only those studies conducted after 2006 will be included since the HPV vaccine was first licensed in June 2006 [19].
3. Studies emphasizing barriers and enablers for HPV vaccine implementation will be included.
4. Studies emphasizing HPV vaccinations among females will be considered.

Regardless of the quality or rigor of the research, studies that match the inclusion criteria outlined in the prepared table will be chosen. This scoping review will encompass quantitative, qualitative, and mixed-methods studies, while excluding reviews, comments, and conference proceedings from the scope. Additionally, the reference lists of included papers will be reviewed to ensure that all relevant literature will be included (Table 2).

**Table 2:** Inclusion and exclusion criteria

Inclusion criteria	Exclusion criteria
Studies focused on factors related to HPV vaccine implementation	Investigations are focusing on cervical cancer treatment
Studies focus on the implementation/introduction process of HPV vaccination	Studies focused on vaccine efficacy rather than HPV vaccine implementation
All available studies were conducted in South Asia	This study will exclude reviews, comments, study abstracts and conference proceedings
Studies published in the English language, 2006 onwards	
This study will only include original studies, such as quantitative, qualitative, and mixed-methods studies	

### Stage 4: Charting the data

Data charting will be carried out with the help of a pre-defined data charting format. Data charting for each paper incorporated in the review aimed to be conducted by three reviewers independently using Rayyan. For any disagreement, another reviewer’s opinion will be sought. The data charting will include details about the participants, context, concept, study methods, facilitators, barriers, and critical relevant findings to the review questions. To fulfil these objectives, data extraction for included studies will be carried out. The data extraction form will include the following details in the study, namely, (i) title, (ii) author, (iii) publication year, (iv) study setting, (v) aims and objective (vi) country where the research was conducted, (vii) design, (viii) participants, (ix) results, (x) findings pertinent to the query, (xi) conclusion, and (xii) recommendations. Along with these retrieved data, narrative data will be gathered from the following areas: the countries’ vaccination implementation methods and guidelines and the reasons that obstruct implementation. The authors of the papers will be contacted if necessary to obtain any additional or missing information. This procedure should aid in the identification of gaps in the research area.

### Stage 5: Collating, summarizing, and reporting the results

The evidence generated through this study will be presented in the form of narrative synthesis. We will use various tables to sum up the characteristics and findings of the studies that were included. To identify the literature that has been generated since June 2006, we will compile a list of South Asian countries and sources from which HPV vaccine research has been taken. By collecting quantitative and qualitative information from the established data charting table, we will map the available literature by reporting studies that characterize the HPV vaccine implementation and highlight the factors influencing the implementation of HPV vaccination in South Asia. The second goal of the study will be to outline the lessons learnt and empirical evidence from South Asian nations on implementing the HPV vaccination at scale. In countries where the HPV vaccination is insufficient, these experiences can be used as evidence to help implement the HPV vaccination effectively. Finally, the findings will be compiled into a cohesive publication with scientific and clinical implications.

## Discussion

The present scoping review seeks to map the evidence and experiences on the implementation of HPV vaccination. The evaluation is expected to uncover the constraints and opportunities affecting the implementation of the HPV vaccine. The adoption of vaccine in India and similar countries can be aided by lessons learnt from the South Asian nations where it has been implemented. These lessons can serve as well-informed empirical data. Additionally, the forthcoming discussion of the study manuscript will encompass engagement with stakeholders in HPV vaccination, enriching the report’s findings. By highlighting the shortcomings and successes in introducing the HPV vaccine at a large scale in these countries, the report’s findings are anticipated to benefit the public health system. Thus, by filling in the gaps and emphasising the positives, south Asian countries where HPV vaccine is not implemented can learn successful implementation methods from similar nations and improve its own vaccination techniques to expand the immunization campaign generally.

## Data Availability

Data sharing is not applicable to this study.

## Abbreviations

HPV: Human papillomavirus
LMICs: Low- and middle-income countries
WHO: World Health Organization
PRESS: Peer Analysis of Electronic Search Strategies
PRISMA: Preferred Reporting Items for Systematic Review and Meta-Analysis
PRISMA-ScR: Preferred Reporting Items for Systematic Review and Meta-Analysis extension for scoping reviews
PRISMA-P: Preferred Reporting Items for Systematic Review and Meta-Analysis extension for protocols
